# The Impact of Biologic Treatment on PD-1/PD-L1 Pathway Disturbances in Psoriasis

**DOI:** 10.3390/jcm12134179

**Published:** 2023-06-21

**Authors:** Michał Adamczyk, Joanna Bartosińska, Dorota Raczkiewicz, Anna Michalak-Stoma, Dorota Krasowska

**Affiliations:** 1 Department of Dermatology, Venereology and Pediatric Dermatology, Medical University of Lublin, 20-081 Lublin, Poland; jbartosinski@gmail.com (J.B.); dor.krasowska@gmail.com (D.K.); 2 Department of Cosmetology and Aesthetic Dermatology, Medical University of Lublin, 20-093 Lublin, Poland; 3 Department of Medical Statistics, School of Public Health, Center of Postgraduate Medical Education, 01-826 Warsaw, Poland; dorota.raczkiewicz@cmkp.edu.pl

**Keywords:** psoriasis, systemic treatment, biologic treatment, immune checkpoint

## Abstract

The programmed death-1 (PD-1) receptor plays a major physiological role in the maintenance of immune tolerance and, by interaction with its ligands (PD-L1 and PD-L2), prevents the development of multiple immune-mediated diseases. There is growing evidence of the PD-1/PD-L1 pathway playing an important role in the pathogenesis of psoriasis. In total, 84 subjects with psoriasis were included in this study, together with 29 healthy subjects as a control group. Twenty-eight of the psoriatic patients were treated with biologic therapy (TNF-alpha, interleukin (IL)-12/23, or IL-17 inhibitors). The amounts of PD-1- and PD-L1-positive T-cells in peripheral blood were evaluated using flow cytometry. Significantly lower levels of peripheral blood mononuclear cells (PBMCs) with the expression of PD-1 and PD-L1 were found in psoriatic patients compared to healthy individuals, i.e., CD3/PD-1-, CD3/PD-L1-, CD4/PD-1-, CD4/PD-L1-, CD8/PD-L1-, CD19/PD-1-, and CD19/PD-L1-positive cells. Biologic treatment resulted in the elevation of CD3/PD-L1- and CD8/PD-L1- and a decrease in CD8/PD-1-positive PBMCs. Our results confirm previous observations of the PD-1/PD-L1 pathway being disrupted in psoriasis, and that these disturbances may play an important role in development of the disease. Biologic drugs may reverse several abnormalities observed within this pathway, which may explain their excellent efficacy in the treatment of psoriasis. Further research should be conducted to fully explain the results obtained.

## 1. Introduction

Psoriasis is a chronic, immune-mediated disease which manifests with clinically typical, erythematous, scaling papules, and plagues that may appear anywhere on the skin surface. The understanding of psoriasis as an only cutaneous problem changed completely in recent decades; now it is considered a systemic condition with multiple associated problems, including psoriatic arthritis, metabolic syndrome, and atherosclerosis with its complications [1].

The pathogenesis of psoriasis is complex and still not fully understood. The appearance of psoriatic plagues on the skin results from the pathological activity of several subtypes of lymphocytes, including Th1 and Th17 secreting proinflammatory cytokines, i.e., IFN-γ, TNF-α, and IL-17. Disruption of mechanisms, ensuring immunotolerance, is thought to play an important role in the activation of the mechanisms in psoriasis, as well as in other immune-mediated diseases [2,3]. Programmed death-1 (PD-1) is an inhibitory co-receptor encoded by the PDCD1 gene and expressed on various white blood cells. Via interaction with its ligands (PD-L1 and PD-L2), which are present on multiple peripheral cells, it mediates self-immunotolerance, in particular, by the reduction in CD4+ T-cells activity and the induction of T-regulatory lymphocytes (Tregs) [2,4].

Therapy of various neoplasms with monoclonal antibodies inhibiting PD-1 may result in new-onset psoriasis or the exacerbation of pre-existing psoriatic skin lesions. Boenigen et al. described 21 of such cases during treatment of lung cancer with anti-PD-1 agents: nivolumab and pembrolizumab [5]. Bartosińska et al. demonstrated that in patients with psoriatic arthritis (PsA), the levels of circulating CD4+ PD-1+ and CD8+ PD-1+ T-cells were significantly decreased compared to healthy control subjects [6]. In peripheral blood mononuclear cells (PBMCs), the expression of the PDCD1 gene was also lower in patients with psoriasis than in the healthy control group [7]. These results seem to be reasonable, for lack of PD-1 activity may lead to the sustained activation of T-cells, the production of inflammatory cytokines, and, as a consequence, the development of psoriatic skin lesions. Recent work by Tanaka et al. proved an important role of PD-L1 expressed on Langerhans cells (LCs) in a murine model of imiquimod-induced psoriasis-like dermatitis. In wild-type mice, imiquimod application induced the expression of PD-L1 on LCs in both ear skin and skin-draining lymph nodes. In conditional knockout mice lacking PD-L1 expression on LCs, there was significantly more severe imiquimod-induced psoriasis-like dermatitis than in wild-type littermates [8].

Biologic drugs have revolutionized the approach to patients suffering from moderate-to-severe forms of plaque psoriasis since their first approval at the beginning of the 21st century. By the inhibition of selected cytokines playing key roles in the immunopathogenesis of psoriasis, they are characterized by a better efficacy and safety profile than standard systemic therapies [9].

In this study, we sought to evaluate the expression of PD-1 and PD-L1 on different types of PBMCs in patients with psoriasis and healthy controls. Another aim was to determine whether and how the treatment of psoriasis with biologic therapies impacts the expression of inhibitory co-receptor PD-1 and its ligand on PBMCs as no such studies have been published to date.

## 2. Materials and Methods

### 2.1. The Study Group

Eighty-four subjects with chronic plaque psoriasis treated in the Department of Dermatology, Venerology and Paediatric Dermatology, Medical University of Lublin, Poland, were qualified to the study. Inclusion criteria were as follows: age ≥18 years, active psoriatic skin lesions, and >1 year of disease duration. Subjects with other types of psoriasis (i.e., erythrodermic, pustular, and guttate), concomitant immune-mediated diseases, neoplasms, and currently undergoing systemic anti-psoriatic treatment were excluded from the study. Detailed clinical characteristics of the study subjects are presented in Table 1. In 28 of the studied subjects with psoriasis, blood samples were collected twice: before and during biologic therapy for psoriasis (not earlier than 16 weeks after therapy was introduced). The control group included 29 subjects who were not suffering from psoriasis and any other immune-mediated conditions.

Among the subjects treated with biologic drugs, 12 received adalimumab, 6 secukinumab, 5 ustekinumab, 3 ixekizumab, 1 risankizumab, and 1 infliximab. All patients who qualified for biologic therapy must have fulfilled the following criteria: have initial PASI score > 18, BSA > 10, and DLQI > 10; moreover, they must have been treated with at least two standard systemic therapies for their psoriasis without response or with unacceptable side effects.

The study was approved by the Bioethics Committee of the Medical University of Lublin (KE-0254/165/2017). Written informed consent was obtained from each study subject before their inclusion into the study.

### 2.2. Assessment of PD-1, PD-L1

The material used for the study was peripheral blood collected from an ulnar vein in 30 mL quantities into tubes containing EDTA. The collected blood was immediately used to perform a lymphocyte immunophenotype evaluation. 

After the collection, the samples were incubated for 20 min in the dark at room temperature. In the next step, 2 mL each of erythrocyte lysing liquid lysing solution (BD Pharmingen, Franklin Lakes, NJ, USA) was added to the tubes. PBMCs were isolated using density gradient centrifugation on Ficoll-Hypaque (Biochrom AG, Berlin, Germany). Interphase cells were removed, washed twice in phosphate-buffered saline (PBS) without Ca^2+^ and Mg^2+^, and then suspended again in RPMI 1640 containing 2% human albumin. The viability of the obtained PBMCs was determined by tryptan blue staining. A cell viability of less than 90% was taken as a criterion to disqualify the sample from further testing. A Neubauer chamber was used to count viable cells. Cells sized 5 × 10^5^ were incubated for 20 min at room temperature with fluorochrome-labeled monoclonal antibodies. 

Table 2 shows the set of monoclonal antibodies and fluorochromes used to perform lymphocyte surface antigen expression studies. The immunophenotype of peripheral blood cells was assessed using an FACSCalibur flow cytometer (Becton Dickinson, Franklin Lakes, NJ, USA) with a 488 nm argon laser. Acquisition and analysis of the results were performed using CellQuest software (Becton Dickinson, Franklin Lakes, NJ, USA). The CaliBRITE calibration kit (Becton Dickinson, Franklin Lakes, NJ, USA) was used to optimize the flow cytometer settings.

Antibodies were added to 4 cytometric tubes in the following combinations:

1. Anti-CD3 FITC/anti-CD274 PE/anti-CD279 APC.
2. Anti-CD4 FITC/anti-CD274 PE/anti CD279 APC.
3. Anti-CD8 FITC/anti-CD274 PE/anti CD279 APC.
4. Anti-CD19 FITC/anti-CD274 PE/anti CD279 APC.

**Figure 1:**
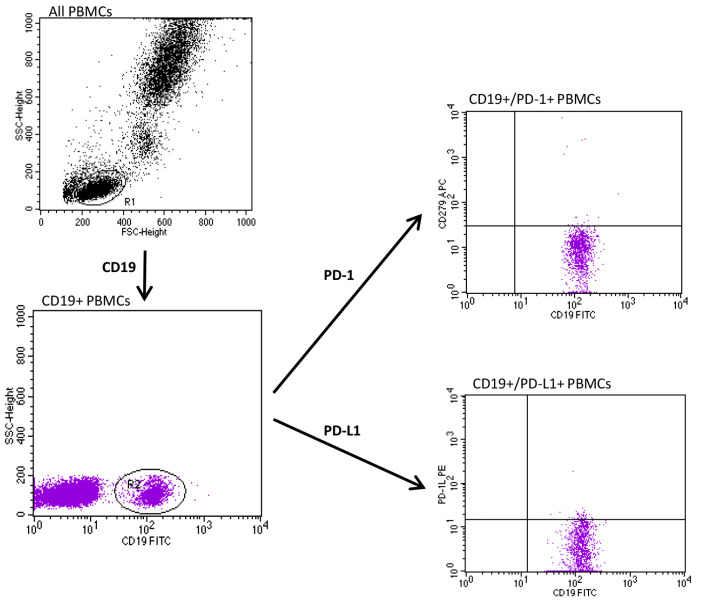
Full gating strategy for flow cytometry analysis of PD-1 and PD-L1 expression on CD19+ T peripheral blood mononuclear cells (PBMCs) in psoriatic subject.

**Figure 2:**
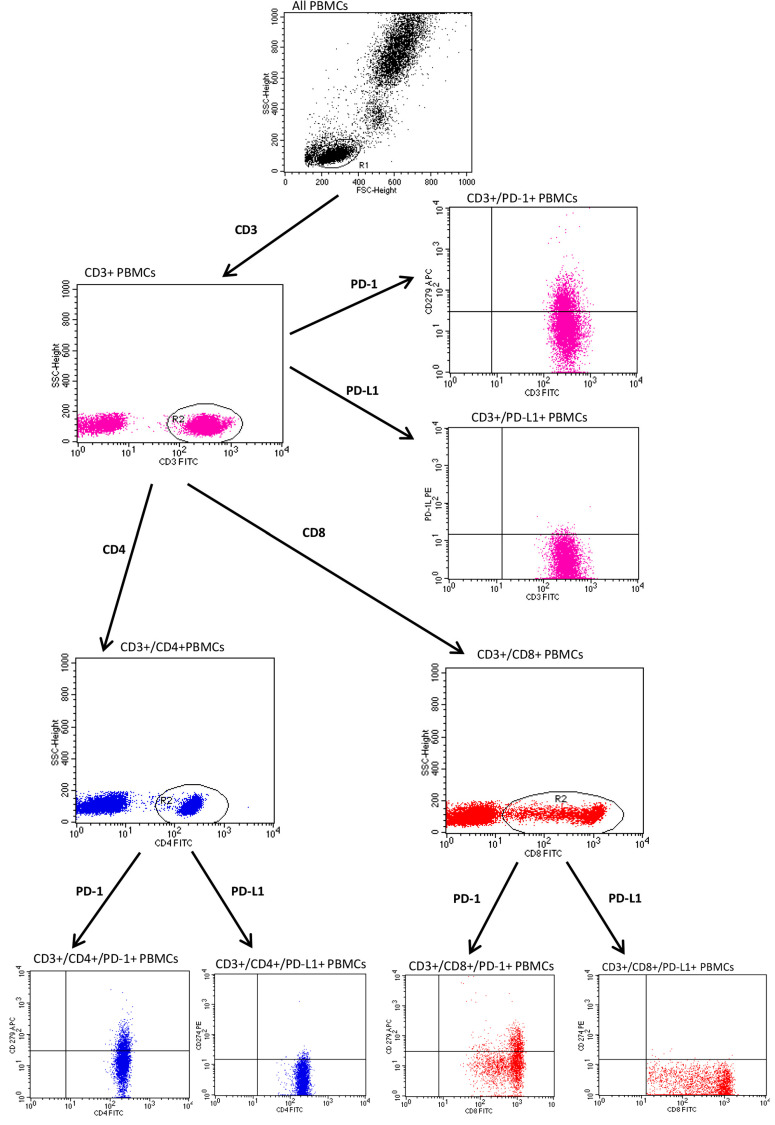
Full gating strategy for flow cytometry analysis of PD-1 and PD-L1 expression on CD3+ T peripheral blood mononuclear cells (PBMCs) in psoriatic subject.

Appropriate forward and side scatter parameters were set to identify and gate lymphocytes from each studied subject. The full gating strategy for the flow cytometry analysis of the PD-1 and PD-L1 expression on PBMCs cells from a psoriatic patient is presented in Figure 1 and Figure 2.

**Figure 3:**
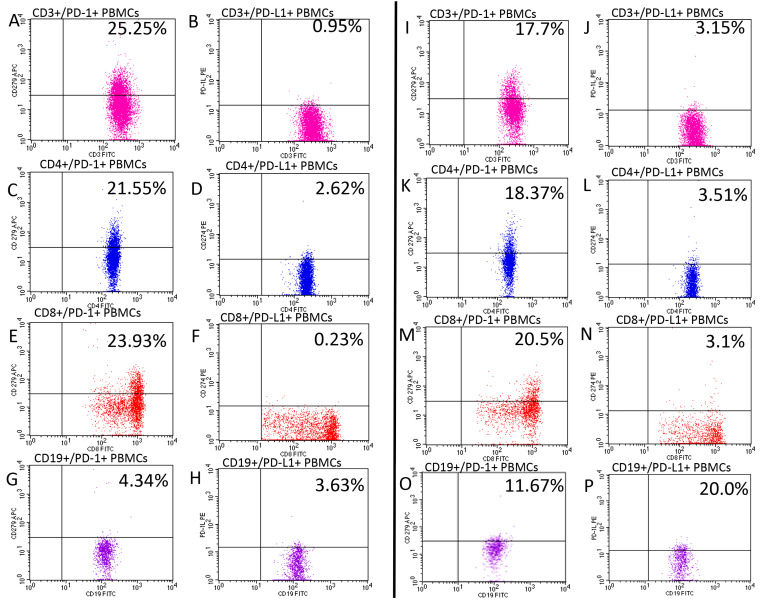
Representative flow cytometry analysis of PD-1 (**A**,**C**,**E**,**G**,**I**,**K**,**M**,**O**) and PD-L1 (**B**,**D**,**F**,**H**,**J**,**L**,**N**,**P**) expression in patients with psoriasis (**A**–**H**) and healthy controls (**I**–**P**). Pink color—CD3+ T-cells, blue—CD4+ T-cells, red—CD8+ t-cells, and purple—CD19+ cells.

The results of the cytometric analysis were presented as the percentage of cells stained with fluorescent-dye-conjugated monoclonal antibodies and as mean fluorescence intensity (MFI), which is an exponent of the amount of expression of a given antigen on the cell surface. Representative flow cytometry analysis of PD-1 and PD-L1 expression in patients with psoriasis and healthy controls is presented in Figure 3.

### 2.3. Statistical Methods

STATISTICA 13.1 software (STATSOFT, Kraków, Poland) was used for the statistical analyses.

We estimated median and interquartile range (IQR) for continuous variables or absolute numbers (n) and relative numbers (%) of the occurrence of items of categorical variables. 

The U Mann–Whitney test was used to compare the age and percentage rates of each PBMC subset between psoriatic patients and the control group. In psoriatic patients before and during biologic treatment, we applied the Wilcoxon test to compare differences between the levels of each PBMC subsets. The Spearman correlation coefficient was used to correlate the PD-1 and PD-L1 expression on different PBMC with PASI, BSA, and duration of psoriasis.

In all the statistical tests, a *p*-value less than 0.05 was interpreted as statistically significant.

## 3. Results

Significantly lower concentrations of CD3/PD-1-, CD3/PD-L1-, CD4/PD-1-, CD4/PD-L1-, CD8/PD-L1-, CD19/PD-1-, and CD19/PD-L1-positive PBMCs were present in subjects with psoriasis compared to the healthy control group (Table 3).

With regard to PASI, BSA, and duration of psoriasis, a positive correlation between the expression of CD3/PD-1 and CD4/PD-1 and duration of disease was found; the longer the duration of psoriasis, the higher the CD3/PD-1 and CD4/PD-1 expression was. Moreover, we revealed a weak correlation between CD19/PD-L1-positive PBMCs and BSA (Table 4).

In psoriatic patients treated with biologic drugs, the levels of CD3/PD-L1- and CD8/PD-L1-positive PBMCs were significantly higher, and the CD8/PD-1-positive PBMC was significantly lower than before therapy. No differences were found between CD4- and CD19-positive cells with the PD-1 and PD-L1 expression before and during biologic treatment (Table 5).

## 4. Discussion

Despite a lot of research being conducted, the exact cause of an enhanced immune response in psoriasis remains unknown. The proper function of PD-1/PD-L1 is crucial in maintaining peripheral immunotolerance in health conditions [4]. A suppressive effect on the immune response is achieved by an interaction between PD-1 and its ligands, PD-L1, and PD-L2, expressed on various, hematopoietic, and non-hematopoietic cells. PD-1/PD-L ligation leads to a reduction in CD4+ T-cells activity and, importantly, an up-regulation in the amounts and function of T-regulatory lymphocytes (Tregs). Tregs are a subtype of T-cells responsible for the suppression of immune response and maintenance of immunotolerance [2].

PD-1 is classified among markers of T-cell activation—its expression was found to be increased in T-cells in various immune-mediated diseases, including systemic lupus erythematosus, rheumatoid arthritis, and psoriasis arthritis [10,11]. An increase in PD-1 expression seems to be inconsistent in inflammatory diseases, due to its inhibitory properties, but it is known that the functioning of the PD-1/PD-L1 pathway is modulated in the conditions of chronic inflammation [2], i.e., it was demonstrated that CD4+ T-cells derived from the synovial fluid of subjects with rheumatoid arthritis require higher concentrations of PD-L1 to achieve inhibition when compared with peripheral blood T-cells [12]. This may be explained by the increased concentration of soluble PD-1 (sPD-1), which inhibits proper PD-1/PD-L1 ligation by binding to PD-L1 on immune cells [2].

In the context of psoriasis and PD-l/PD-L1, the largest quantities of data from the literature refer to frequently observed cases of new onset or worsening of psoriasis in subjects treated for malignant tumors with anti-PD-1 biologic drugs, in particular, nivolumab and pembrolizumab [5]. Most of these cases are mild and can be sufficiently controlled with topical agents, not being a reason for treatment cessation [13].

In a murine model of imiquimod-induced psoriasis, Imai et al. proved that in PD-1 gene knock-out mice, there was more severe keratinocyte hyperproliferation, higher levels of neutrophils in inflammatory infiltrates, and the expression of higher levels of Th17 cytokines than in wild-type mice [14].

Bommarito et al. demonstrated that in psoriatic arthritis (PsA) and rheumatoid arthritis, inflammatory cytokines, including TNF-α, IL-1, and IL-6, increase the levels of soluble PD-1 (sPD-1). sPD-1 binds to PD-1 ligands and prevents proper PD-1/PD-L ligation, disrupting its inhibitory function, which promotes an inflammatory reaction. The authors found that despite the increased expression of PD-1, CD4+ T-cells derived from the peripheral blood and synovial fluid of patients were resistant to PD-1-mediated suppression, and this effect was probably caused by increased levels of sPD-1 [15]. To date, no studies have evaluated the expression of sPD1 and its importance in psoriasis, either in peripheral blood or a psoriatic plague environment.

In line with the above findings, Peled et al. found that the expression levels of PD-1 on circulating T-cells were significantly higher in PsA than in healthy controls; moreover, they inversely correlated with disease activity measured by DAS28. Furthermore, they found a strong correlation between PD-1 expression levels and the number of tender and swollen joints [11].

Bartosińska et al. demonstrated contradictory data. In their studies, they found a decreased expression of the PDCD1 gene in PMBCs derived from subjects with psoriasis compared to healthy controls [7]. The same authors found that absolute and percentage levels of CD4+ PD-1+ and CD8+ PD-1+ T-cells in the peripheral blood of psoriatic patients with and without psoriatic arthritis were significantly decreased compared to healthy individuals [6,16]. Our present work is in agreement with these findings, but we have analyzed a wider range of PBMNCs, including those with the expression of PD-L1. We have showed a significant reduction in PD-1 and PD-L1 expression on the majority of PBMC subsets, CD3+, CD4+, and CD19+, except from CD8/PD1-positive cells [Table 3]. Reduced PD/PD-L1 expression may explain the lack of inhibitory effect of this pathway on the immune response in psoriasis, and this may be responsible for inflammatory response exacerbation and lead to the development of skin lesions.

In another study, Bartosińska et al. did not show correlations between levels of PBMCs expressing PD-1/PD-L1 and clinical characteristics of psoriasis (including PASI, BSA, and duration pf psoriasis) [17]. Our results showed a correlation between CD3/PD-1- and CD4/PD-1-positive cells and duration of disease. In addition, a correlation between the level of CD19/PD-L1 and BSA was found; however, it was not strong [Table 4].

No studies published to date have evaluated the expression of PD-1 and PD-L1 on PBMCs before and during treatment of psoriasis with biologic drugs. Our data show that biologic drugs with different mechanisms of action have an impact on several of these cells as treatment resulted in a significant increase in CD3/PD-L1- and CD8/PD-L1-positive PMNCs and a significant decrease in CD8/PD-1-positive PBMCs [Table 5]. An increase in T-cells expressing PD-L1 may contribute to enhanced PD-1/PD-L1 ligation and the induction of immune tolerance mechanisms. Interestingly, the only subset of examined PBMCs that decreased after biologic treatment (CD8/PD-1-positive) was that only one was not found to be different between psoriatic patients and healthy controls in the current study [Table 3]. The above observations may justify the superior efficacy of biologic therapies in psoriasis treatment.

The limitations of the present study include that there was a relatively small number of subjects included, and that evaluations were performed on peripheral blood only and not in psoriatic-changed skin. We did not perform separate analyses for subjects on different biologic drugs due to the small number of subjects included in the study. Further research should be conducted on a larger population of patients with respect to different therapies received.

## 5. Conclusions

By showing alterations in the majority of PBMCs expressing PD-1 and PD-L1, our results confirm that PD-1/PD-L1 pathway disturbances are present in subjects with psoriasis and may play an important role in the development of the disease. Biologic therapy may reverse the observed abnormalities, resulting in the elevation of PD-L1 expressing CD3+ and CD8+ lymphocytes and a decrease in CD8/PD-1-positive lymphocytes. These findings may explain their excellent efficacy in the treatment of disease. Further research is needed into larger populations to fully explain the results observed.

## Figures and Tables

**Table 1:** Characteristics of the study subjects with plaque psoriasis (*n* = 84) and control group.

Characteristics	Unit or Category	Results
**Patients with psoriasis:**		
Age, min–max, median (IQR)	Years	18–68, 43 (34–52)
Gender, *n* (%)	Male	60 (71.4)
	Female	24 (28.6)
Weight, min–max, median (IQR)	kg	47–125, 85 (74–95)
BMI, min–max, median (IQR)	kg/m^2^	17.72–46.88, 27.51 (24.44–31.25)
Smoking status, *n* (%)	Non-smokers	30 (38.0)
	Smokers	54 (62.0)
Psoriasis type, *n* (%)	I (the onset <40 years old)	72 (88.1)
	II (the onset >40 years old)	12 (11.9)
Duration of psoriasis, min–max, median (IQR)	Years	1–50, 20 (14–26)
PASI, min–max, median (IQR)		5.5–47.0, 18.0 (13.1–21.3)
BSA, min–max, median (IQR)		6–80.0, 23.3 (15.0–39.8)
PsA, *n* (%)	Yes	27 (30.9)
**Control group:**		
Age, min–max, median (IQR)	Years	24–65, 40 (33–52)
Gender, *n* (%)	Male	20 (69.0)
	Female	9 (31.0)

IQR—interquartile range.

**Table 2:** Set of monoclonal antibodies and fluorochromes used to perform lymphocyte surface antigen expression studies.

Specificity	Fluorochrome	Producer	Clone	Isotype
Mouse anti-human-CD3	FITC	BD Biosciences, Franklin Lakes, NJ, USA	MEM-57	Mouse IgG2a, κ
Mouse anti-human-CD4	FITC	BD Biosciences, Franklin Lakes, NY, USA	RPA-T4	Mouse IgG1, κ
Mouse anti-human-CD8	FITC	BD, Biosciences, Franklin Lakes, NY, USA	SK1	Mouse BALB/c IgG1, κ
Mouse anti-human-CD19	FITC	BD Biosciences, Franklin Lakes, NY, USA	HIB19	Mouse IgG1, κ
Mouse anti-human-CD279	APC	BD Biosciences, Franklin Lakes, NY, USA	MIH4	Mouse IgG1, κ
Mouse anti-human-CD274	PE	BD Biosciences, Franklin Lakes, NY, USA	MIH1	Mouse BALB/c IgG1, κ

**Table 3:** A comparison of percentage rates of PBMCs expressing PD-1 and PD-L1 between psoriatic patients (*n* = 84) and control group (*n* = 29).

PBMC Subtype	Psoriasis	Control	*p*
	Median (IQR)	Median (IQR)	
**CD3/PD-1**	**13.78 (11.03–18.42)**	**17.26 (14.51–21.00)**	**0.021**
**CD3/PD-L1**	**1.23 (0.74–2.72)**	**3.26 (1.67–8.20)**	**<0.001**
**CD4/PD-1**	**13.58 (9.94–17.46)**	**18.10 (13.77–25.25)**	**0.002**
**CD4/PD-L1**	**2.14 (1.14–4.49)**	**4.30 (3.28–5.28)**	**0.001**
CD8/PD-1	13.70 (9.78–17.32)	14.90 (8.80–18.61)	0.576
**CD8/PD-L1**	**0.58 (0.28–1.57)**	**1.72 (0.87–3.10)**	**<0.001**
**CD19/PD-1**	**2.48 (1.44–4.34)**	**10.65 (6.14–12.20)**	**<0.001**
**CD19/PD-L1**	**5.94 (1.39–11.11)**	**10.95 (4.67–20.00)**	**0.002**

*p* for U Mann–Whitney test, IQR—interquartile range. Statistically significant differences in bold.

**Table 4:** Correlations of PD-1 and PD-L1 expression on different PBMC with PASI, BSA, and duration of psoriasis.

PBMC Subtype	PASI		BSA		Duration of Psoriasis	
	r	*p*	r	*p*	r	*p*
CD3/PD-1	−0.101	0.361	−0.132	0.233	**0.261**	**0.018**
CD3/PD-L1	−0.018	0.873	0.069	0.532	−0.080	0.473
CD4/PD-1	−0.033	0.767	−0.039	0.727	**0.263**	**0.017**
CD4/PD-L1	−0.008	0.944	0.075	0.500	−0.138	0.216
CD8/PD-1	−0.149	0.177	−0.087	0.429	0.200	0.072
CD8/PD-L1	0.094	0.393	0.062	0.574	−0.011	0.925
CD19/PD-1	−0.096	0.384	0.043	0.697	0.203	0.068
CD19/PD-L1	−0.014	0.898	**0.244**	**0.025**	−0.063	0.576

r—Spearman correlation coefficient; statistically significant differences in bold.

**Table 5:** A comparison of percentage rates of PBMCs expressing PD-1 and its ligands in psoriatic patients before and during treatment with biologic drugs (*n* = 28).

PBMC Subtype	Before Treatment	During Treatment	*p*
	Median (IQR)	Median (IQR)	
CD3/PD-1	13.59 (10.08–20.77)	13.72 (9.32–16.24)	0.078
**CD3/PD-L1**	**1.33 (0.92–2.36)**	**3.74 (1.85–11.11)**	**0.002**
CD4/PD-1	15.64 (10.00–17.69)	12.11 (9.78–16.32)	0.471
CD4/PD-L1	2.11 (1.43–3.37)	3.1 (1.42–6.03)	0.428
**CD8/PD-1**	**14.73 (10.04–21.10)**	**12.84 (7.00–15.73)**	**0.041**
**CD8/PD-L1**	**0.59 (0.28–1.36)**	**1.65 (0.63–5.84)**	**0.006**
CD19/PD-1	3.74 (1.79–4.36)	2.91 (1.14–6.11)	0.737
CD19/PD-L1	4.77 (1.77–10.00)	9.6 (2.67–13.39)	0.302

*p* for Wilcoxon rank test, IQR—interquartile range. Statistically significant differences in bold.

## Data Availability

All data presented in this study are reported in this manuscript.
